# Passive Coping Strategies Are Associated With More Impairment In Quality Of Life In Patients With Fibrous Dysplasia

**DOI:** 10.1007/s00223-018-0441-1

**Published:** 2018-06-13

**Authors:** M. Rotman, C. D. Andela, B. C. J. Majoor, P. D. S. Dijkstra, N. A. T. Hamdy, A. A. Kaptein, N. M. Appelman-Dijkstra

**Affiliations:** 1 Division of Endocrinology, Department of Medicine, Center for Bone Quality, Leiden University Medical Center, Leiden, The Netherlands; 2 Department of Orthopedic Surgery, Center for Bone Quality, Leiden University Medical Center, Leiden, The Netherlands; 3 Department of Medical Psychology, Center for Bone Quality, Leiden University Medical Center, Leiden, The Netherlands; 4 Department of Internal Medicine, LUMC Center for Bone Quality, Leiden University Medical Center, Albinusdreef 2, P.O. Box 9600, 2300 RC Leiden, The Netherlands

**Keywords:** Coping strategies, Fibrous dysplasia, McCune Albright syndrome, Quality of life

## Abstract

Impairments in quality of life (QoL) have been reported in patients with fibrous dysplasia (FD). Here, we examine coping strategies in FD and assess whether these coping strategies are associated with QoL and disease severity. Ninety-two patients (66% females) filled out the Utrecht Coping List (UCL), Short Form-36, and the Brief Pain Inventory (BPI). Coping strategies of patients with FD were compared with reference data from a random sample of Dutch women and patients with chronic pain. Compared to healthy adults, patients expressed more emotions (*p* < 0.01). Compared to patients with chronic pain, patients with FD used more active coping strategies (*p* < 0.001), and sought more distraction (*p* = 0.01) and more social support (*p* < 0.001). Using more passive coping strategies was associated with more impairment in social function, physical role, mental health, vitality (all *p* < 0.001), and general health (*p* < 0.01). Using more avoidant coping strategies was associated with worse mental health and less vitality (both *p* < 0.01). More expression of emotions was associated with worse mental health (*p* < 0.01). Type and clinical severity of FD were not associated with coping behavior. Patients with FD have different coping strategies compared to random Dutch reference populations with or without pain. In FD, using more passive coping strategies was associated with more impairment in several aspects of QoL. There was no relationship between coping behavior and clinical characteristics, pointing to biomedical variables not determining the way patients cope with their illness. Recognition of less effective coping strategies can be helpful in the understanding and adaptation of these coping strategies, improving personalized clinical care, with the ultimate goal to improve QoL in patients with FD.

## Introduction

In a recent study, it was shown that patients with fibrous dysplasia (FD), particularly those patients with bone pain, reported significant impairment in quality of life (QoL), and that disease severity, as reflected by higher skeletal burden scores (SBS) and elevated serum bone turnover markers, was associated with more severe impairment in QoL [1]. According to the Common-Sense Model of self-regulation (CSM), QoL is influenced by two major determinants: illness perceptions and coping strategies [2, 3]. The CSM provides a framework for describing the way by which patients manage the perceived threat of their illness using coping behavior. The CSM essentially consists of three stages: The first stage, the Illness perceptions stage, is further divided into five categories: identity, cause, timeline, consequences, and cure. The second stage consists of coping strategies that are developed based on illness perceptions. In a previous study of our department, it was demonstrated that illness perceptions of patients with FD differed significantly from patients with acute or chronic pain due to other medical conditions [4]. The third stage of the CSM consists of the effect of these coping strategies on the patient’s QoL.

Coping refers to the skills used in handling situations, crises, or life events, with coping strategies influencing the way an individual handles the burden of having a chronic illness. The skills a person possesses to cope with stressful situations are not set at birth but are known to develop during early childhood [5]. Coping skills include emotional, behavioral, and cognitive responses. The method described by Lazarus, which is often used to structure coping strategies, entails grouping coping strategies into three categories [6]. The first category is ‘problem-directed coping,’ which involves directly attempting to change a problematic situation. The second category is ‘appraisal centered coping,’ which aims at changing the threatening character of an event, thereby changing the perception of a situation. This mainly involves cognitive and observational functions, such as reassuring thoughts applied to comfort oneself in a difficult situation. The third category of coping strategies aims at reducing arousal, and is described by Lazarus as *‘*symptom-directed palliative modes of coping.’ Examples of this way of coping are smoking or drinking alcohol to relieve an experienced tension. Several studies using the CSM to examine coping strategies in patients with chronic illnesses have identified the significant impact of these strategies on QoL [7–10]. To the best of our knowledge, there have been no studies that examined coping strategies in patients with FD. Therefore, the aim of the present study was to examine coping strategies in patients with FD [2, 11]. For this evaluation, coping strategies of patients with FD were compared with coping reference data of a random Dutch sample and a group of patients with chronic pain. Considering the previous observed illness perceptions and impairment in QoL, it was hypothesized that patients with FD report less effective coping strategies compared to healthy control data. The second aim was to assess whether coping strategies in patients with FD correlate with QoL and clinical severity. Considering the theory of the CSM, it was hypothesized that less effective coping strategies correlate with more impairment in QoL.

## Patients and Methods

### Procedure

**Fig. 1:**
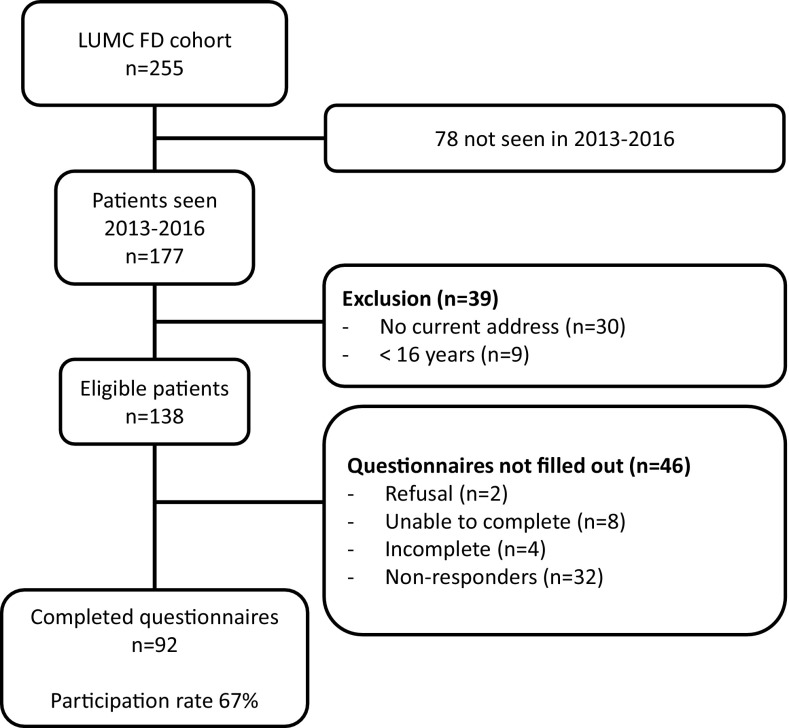
Flowchart of patient inclusion and participation rate

As part of the protocol used in our ongoing study addressing various aspects of QoL in patients with FD [1, 4], three different questionnaires were sent to 255 consecutive patients with a confirmed diagnosis of FD, aged ≥ 16 years, who attended the outpatient clinic of the Centre for Bone Quality of the Leiden University Medical Center between 2013 and 2016. Ninety-two of 138 eligible patients completed the questionnaires, representing a response rate of 67% (Fig. 1). Data on age, gender, age at diagnosis of FD, FD type [monostotic FD (MFD), polyostotic FD (PFD), and the McCune Albright syndrome (MAS)], skeletal burden scores (SBS), alkaline phosphatase (AP), fracture history, and history of previous surgical intervention were retrieved from the patients’ medical records. The diagnosis of MAS was established on the basis of PFD associated with endocrinopathies in the form of precocious puberty, with occasional additional endocrinopathies such as growth hormone or prolactin excess or hyperthyroidism. The presence of cafe-au-lait patches was recorded, but not considered essential for the diagnosis of MAS [12]. The UNESCO International Standard Classification of Education [13] was used to classify the patients’ level of education. Low level of education was defined as a primary to lower secondary education; medium level of education was defined as an upper secondary to post-secondary non-tertiary education; and high level of education was defined as the first and second stage of tertiary education. Two of the authors blindly assessed SBS in all patients with available ^99m^Technetium skeletal scintigraphy [14].

### Questionnaires

Questionnaires used were the Utrecht Coping List Questionnaire (UCL) to evaluate coping strategies, and the Short Form-36 (SF-36) and Brief Pain Inventory (BPI) questionnaires to evaluate QoL [15–18]. For a detailed description of the SF-36 and BPI please refer [1]. The UCL is a widely used validated questionnaire to study coping strategies, which is based on the assumption that individuals prefer a certain way of coping, regardless of the situation: the coping strategy. The UCL consists of 47 statements covering seven domains, each representing a different coping strategy. Each statement is scored on a four-point scale ranging from 1 (seldom or never) to 4 (very often). The seven domains have a different number of statements, so that the individual subscales have different maximum scores [19]. *Active coping*, the first domain, consists of seven items (scores ranging from 7 to 28) assessing a person’s ability to become aware of different points of view in addressing a problem, while confidently intending to solve it. *Palliative coping* (eight items, range 8–32) refers to not having to deal with a problem by distracting oneself with other occupations, such as smoking or drinking alcohol. *Avoidant coping* (eight items, range 8–32) entails not facing the problem and preferably attending as little as possible to an issue. *Seeking social support* (six items, range 6–24) assesses a person’s tendency to ask for help or comfort and understanding from family and/or friends. The domain *passive coping* (seven items, range 7–28) covers having a negative attitude towards the problem, feeling overwhelmed by it, or worrying about past experiences. The sixth domain, *Expressing Emotions* (three items, range 3–12), refers to someone’s tendency to show emotions as anger or fear. Lastly, *Fostering Reassuring Thoughts* (five items, range 5–20) refers to holding on to a positive attitude towards the problem, believing there are worse things in life [19].

### Reference Populations

Coping strategies of patients with FD were compared with two reference populations [19, 20]. The first reference population consisted of two population samples; a population of randomly selected Dutch women and a group of Dutch female nurses, bringing a combined total reference population of 107 women with a mean age of 55 years (range 45–65 years) [19]. The second reference population consisted of a group of patients with chronic pain due to an unknown cause, localized in the hip and/or knee and reported to have occurred on at least three occasions over the previous month [21]. Patients were asked to answer the following set of questions: ‘Did you have any pain or other complaints about your joints in the past month?’ (yes or no) and ‘can you point out the painful joints?’. This population included 15 men and 44 women with a mean age of 64 years (range 55–74 years).

### Statistical Analysis

SPSS for Windows, version 24.0 (SPSS, Inc., Chicago, IL, USA) was used for statistical analysis. Data are presented as mean (± SD) or as median (intermediate range). Categorical data are presented as percentages. To determine whether coping strategies differed between various types of FD, UCL scores were compared between patients with MFD, PFD, and MAS using an ANOVA. For the comparison of coping strategies of patients with FD and coping strategies of the reference groups, a pooled sample *T*-test was used (with level of significance set at *p* < 0.05). Correlation analyses were performed between coping strategies (UCL) and QoL (SF-36, BPI) and clinical severity of FD (SBS scores and AP levels). Pearson correlations were performed when data were normally distributed, and Spearman’s Rank correlations when data were not normally distributed. Multiple regression was used for age, age at diagnosis of FD, and educational level to check for predictive factors for coping behavior. Level of significance was set at *p* < 0.01 to correct for multiple testing.

## Results

### Clinical Characteristics (Table 1)

**Table 1:** Patient Characteristics per FD type

	Total (*n* = 92)	MFD (*n* = 63)	PFD (*n* = 22)	MAS (*n* = 7)
Gender (male/female)	31/61	22/41	9/13	0/7
Age Age at diagnosis	47 (16–80) 30 (1–68)	43 (16–80) 31 (3–68)	51 (14–68) 31 (3–61)	52 (42–62) 4 (1–15)
Educational level				
Low	10 (11%)	7 (11%)	3 (14%)	0
Medium	24 (26%)	16 (25%)	7 (32%)	1 (14%)
High	45 (49%)	30 (48%)	9 (40%)	6 (86%)
Unknown	13 (14%)	10 (16%)	3 (14%)	0
Alkaline phosphatase (U/L)	89.9 (47.3)	70.5 (19.3)	117 (62.9)	104.5 (52.7)
Skeletal burden score (%)	8.7 (12.8)	1.6 (1.6)	13.3 (7.8)	33.9 (20.4)
Follow-up (years)	17.05 (14.8)	13.2 (10.4)	20.2 (15.9)	41.6 (20.1)
Precocious puberty	7 (7.6%)	0	0	7 (100%)
Growth hormone excess	2 (2.2%)	0	0	2 (29%)
Hyperprolactinemia	0	0	0	0
Hyperthyroidism	2 (2.2%)	0	0	2 (29%)

Data expressed as median (range), mean (SD) or number and percentage

The median age of the patients [31 male (34%), 61 female (66%)] was 47 years (range 16–80 years). There was a significant difference between FD types in age (MFD 43.5 years, PFD 51.0 years, MAS 52.3 years; *p* = 0.04), in the occurrence of at least one fracture (MFD 13 (21%), PFD 10 (45%), MAS 6 (86%); *p* < 0.001), and in the history of previous surgical intervention for FD (MFD 20 (32%), PFD 11 (50%), MAS 20 (32%); *p* = 0.001). The majority of our cohort reported having pain at the site of the FD lesion (53%), with a mean pain severity score on the BPI of 3.2 (SD 2.6) and pain interference score of 2.2 (SD 1.4).

### Coping Strategies in Patients with FD Compared to the Reference Populations (Fig. 2)

**Fig. 2:**
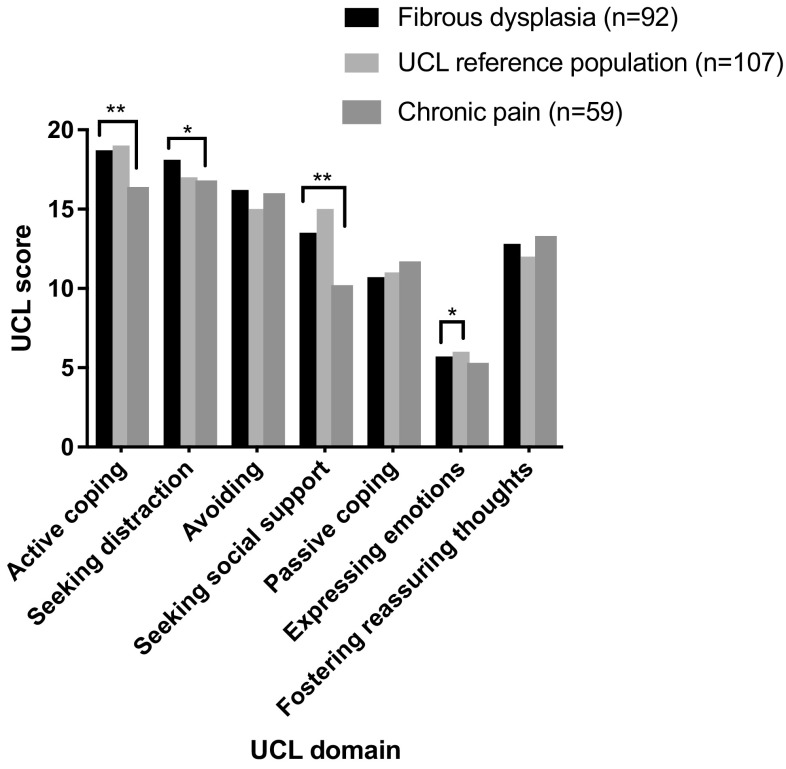
Comparison of UCL scores between FD patients, the UCL reference population, and chronic pain patients. Bar chart comparing the UCL (Utrecht Coping List) scores between FD patients and the UCL reference population consisting of a random sample of Dutch women and between FD patients and patients with chronic pain. Significant differences are illustrated by *p < 0.01 or **p < 0.001

There was no significant difference in coping strategies between subtypes of FD, therefore patients were analyzed as one group. Compared to individuals from a random sample of Dutch women, patients with FD reported expressing less emotions (*p* < 0.01). There were no significant differences in other coping strategies between patients with FD and the reference data of a random sample of Dutch women. Compared to patients with chronic pain, patients with FD reported using more active coping strategies (*p* < 0.001) and seeking more distraction (*p* < 0.01) and social support (*p* < 0.001). There were no significant differences in other coping strategies between patients with FD and patients with chronic pain.

### Correlations Between Coping Strategies and QoL (Table 2)

**Table 2:** Correlations between UCL and SF-36 domains in fibrous dysplasia

	Physical function		Role physical function		Bodily pain		General health		Vitality		Social function		Role emotional		Mental health	
	*r*	*p* value	*r*	*p* value	*r*	*p* value	*r*	*p* value	*r*	*p* value	*r*	*p* value	*r*	*p* value	*r*	*p* value
Active	0.190	0.090	0.082	0.467	0.157	0.141	0.151	0.160	0.169	0.118	0.111	0.301	0.078	0.487	0.235	0.037
Palliative	0.102	0.372	0.039	0.736	− 0.027	0.801	0.005	0.962	0.093	0.395	− 0.009	0.933	0.089	0.434	0.094	0.417
Avoiding	− 0.197	0.078	− 0.108	0.338	− 0.233	0.028	− 0.033	0.763	− 0.318*	**0.003**	− 0.176	0.099	0.058	0.607	− 0.290*	**0.010**
Social	0.050	0.661	0.032	0.774	0.076	0.477	0.150	0.163	0.089	0.414	0.103	0.335	0.062	0.582	0.038	0.742
Passive	− 0.111	0.328	− 0.363**	**0.001**	− 0.348**	**0.001**	− 0.317*	**0.003**	− 0.434**	**0.000**	− 0.446**	**0.000**	− 0.215	0.056	− 0.598**	**0.000**
Express emotions	0.153	0.171	0.114	0.312	− 0.053	0.621	0.009	0.937	− 0.048	0.661	− 0.028	0.795	0.096	0.392	− 0.290*	**0.010**
Reassuring thoughts	− 0.094	0.411	− 0.015	0.894	− 0.016	0.883	0.065	0.550	0.050	0.651	− 0.009	0.933	0.076	0.504	0.113	0.327

Correlations between UCL and SF-36 domains with level of significance set at *p* = 0.01 are given in bold

*p < 0.01, **p < 0.001

More passive coping was associated with a higher reported average and maximum pain and pain interference in daily activities (*r* = 0.32, *p* < 0.01; *r* = 0.29, *p* < 0.01 and *r* = 0.34, *p* ≤ 0.01, respectively) (BPI). Furthermore, using more passive coping correlated with a higher impairment in social function (*r* = − 0.45, *p* < 0.001), physical role (*r* = − 0.36, *p* < 0.001), mental health (*r* = − 0.6, *p* < 0.001), vitality (*r* = − 0.43, *p* < 0.001), pain (*r* = − 0.35, *p* < 0.001), and general health (*r* = − 0.32, *p* < 0.01). Avoidant coping strategies correlated with perceived worse mental health (*r* = − 0.29, *p* < 0.01) and less vitality (*r* = − 0.32, *p* < 0.01). More expression of emotions was associated with perceived worse mental health (*r* = − 0.29, *p* < 0.01) (SF-36).

### Correlations Between Coping Strategies and Clinical Severity

Coping strategies were not correlated with clinical severity of FD (as measured by SBS and AP levels). Age, age at diagnosis of FD, and educational level were not predictive for coping behavior.

## Discussion

The present study demonstrates that patients with FD tend to express less emotions compared to reference data of a random sample of Dutch women. On the other hand, patients with FD reported more active coping, seeking more distraction and more social support compared to patients with chronic pain. There were no differences in coping strategies between the three different types of FD (MFD, PFD, and MAS). Furthermore, using more passive coping strategies and more avoidant coping strategies were associated with more impairments in several aspects of QoL, such as social functioning, mental health, and vitality. There were no associations between coping strategies and clinical severity of FD.

The observation that patients with FD reported more active coping than patients with chronic pain, could potentially be explained by the fact that in the reference group of patients with chronic pain due to an unknown cause, the pain was localized in the hip and/or knee and was reported to have occurred on at least three occasions over the previous month [9], whereas not all patients with FD have complaints of pain (58%). Furthermore, no difference in coping behavior was found between FD patients with and FD patients without pain (data not shown). In addition, seeking social support was used more often by patients with FD than by patients with chronic pain, possibly indicating that a diagnosis of FD comes with a greater sense of social acceptance and greater tendency to ask friends and family for help than is the case with patients with chronic pain due to an unknown cause. In our cohort, higher reported average and maximum pain as well as pain interference in daily activities were associated with more passive coping strategies. The assumption that pain influences coping behavior is not unexpected and has been previously put forward [22, 23]. In accordance with the theory of the CSM, we observed that coping strategies of patients with FD affected their QoL, which is in keeping with observations in patients with other chronic disorders [8].

This study is the first in its sort by addressing coping strategies of a relatively large cohort of patients with FD. Preliminary results of a large QoL study in patients with rare bone diseases show a wide variation in reported QoL of patients with osteogenesis imperfecta (OI), X-linked hypophosphatemia (XLH), and FD [24]. Coping behavior in these patient populations, however, has not been studied previously. Over two-thirds of the patients in our FD population completed the UCL, a potential limitation of this study, however, is the possible gender bias, since 66% of respondents were female. Particularly because it is known that women use different coping strategies than men [19]. In our cohort we did not find a difference in the use of coping strategies based on gender, apart from the known association between female gender and seeking social support as a coping strategy (data not shown). Furthermore, the distribution of the several FD types was somewhat uneven, since the majority had the milder form of monostotic FD, with only 7 of the 92 patients having McCune Albright syndrome (MAS). Therefore, no firm conclusions could be drawn about potential differences in coping strategies between different types of FD. In this study, merely validated QoL questionnaires were used. However, all were generic and domain-specific. For future research it would be interesting to include a disease-specific QoL questionnaire assessing FD-specific aspects of QoL. It has been recommended to combine a disease-specific QoL questionnaire with a generic QoL questionnaire, particularly in rare diseases with a wide spectrum of disease severity such as FD [25]. Unfortunately, a validated disease-specific QoL questionnaire for FD is currently lacking.

In chronic diseases, behavioral factors such as coping behavior are known to potentially influence QoL, as well as disease outcome and even mortality [26]. The beneficial effect of assessing coping strategies and their effect on QoL in clinical care for patient with chronic disease has been previously demonstrated [27]. Therefore, it can be suggested that incorporating the assessment of coping strategies in the clinical care of patients with FD is important for disease outcome and patient well-being.

The present findings show that patients with FD use different coping strategies compared to the general population and patients with chronic pain. In addition, it was shown that less effective coping strategies were associated with more impairment in QoL. It can be postulated that adapting these unfavorable coping strategies into more effective coping strategies will beneficially influence patient perceived QoL. Awareness of treating physicians about these less effective coping strategies in patients with FD will be helpful in the recognition, understanding, and potential adaptation of these coping strategies. Furthermore, referral to a specialized medical psychologist should be considered when inadequate coping strategies are identified. A disease-specific self-management program addressing coping strategies might also be helpful in adapting coping behavior. Such a multidisciplinary approach will improve coping behavior, and thereby QoL and overall disease outcome in patients with FD.
